# Antioxidant Activity of Foods and Natural Products

**DOI:** 10.3390/molecules29081814

**Published:** 2024-04-17

**Authors:** José Pinela, Maria Inês Dias, Carla Pereira, José Ignacio Alonso-Esteban

**Affiliations:** 1 Centro de Investigação de Montanha (CIMO), Instituto Politécnico de Bragança, Campus de Santa Apolónia, 5300-253 Bragança, Portugal; maria.ines@ipb.pt (M.I.D.); carlap@ipb.pt (C.P.); 2 Laboratório Associado para a Sustentabilidade e Tecnologia em Regiões de Montanha (SusTEC), Instituto Politécnico de Bragança, Campus de Santa Apolónia, 5300-253 Bragança, Portugal; 3 Departamento de Ciencias Biomédicas, Facultad de Farmacia, Universidad de Alcalá, Carretera Madrid-Barcelona, Km 33.600, 28871 Madrid, Spain; jignacio.alonso@uah.es

## 1. Introduction

Today, there is growing recognition of the importance of antioxidants in promoting human health and well-being. These compounds play a vital role in protecting the organism from oxidative stress, which is implicated in the pathogenesis of various chronic degenerative diseases, including cardiovascular and neurodegenerative disorders, diabetes, and different types of cancer [1]. Furthermore, antioxidants serve as indispensable agents in food preservation by preventing or slowing down oxidation processes, which could otherwise result in the deterioration of food quality, flavor, color, texture, and nutritional value [2]. This underscores ongoing research endeavors and the emergence of novel applications for antioxidant compounds across different disciplines, including chemistry, food science, nutrition, pharmacology, and medicine.

This Special Issue, titled “Antioxidant Activity of Foods and Natural Products”, has brought together an interesting collection of cutting-edge research and development contributions. A total of 30 manuscripts were submitted for consideration, each undergoing the *Molecules* journal’s rigorous pre-check and peer review process. Ultimately, 12 articles were accepted for publication in this Special Issue, comprising 1 review article and 11 original research articles. These studies address current challenges and topics related to the antioxidant properties of foods and natural products, including the antioxidant activity of plant extracts, botanical preparations, and isolated compounds, the mechanisms of action of antioxidants and pro-oxidants, and their potential therapeutic effects in vitro and in vivo.

## 2. An Overview of Published Articles

Bērziņa and Mieriņa (contribution 1) discussed the role of compounds containing the 1,3-dicarbonyl moiety in preventing oxidative damage and their potential as antioxidants. The study suggested that while some research points to the importance of substituents in the benzene ring, others indicate the crucial role of the 1,3-dicarbonyl moiety itself. Additionally, structural elements such as α-monosubstituted compounds and cyclic structures were identified as important factors influencing antiradical and antioxidant activity. Overall, the findings suggest that 1,3-dicarbonyl compounds hold potential for the development of effective antioxidants.

The study by Altuntaş et al. (contribution 2) explored the phenolic composition and bioactivity of 24 Anatolian propolis samples from Türkiye, aiming to classify them by origin. Phenolic and aromatic acids were identified, and propolis’s antioxidant and antimicrobial properties were highlighted. While no single compound was solely responsible for these effects, the natural mixture as a whole demonstrated therapeutic significance. The study’s use of principal component analysis effectively clustered samples based on their biochemical properties, providing insights into propolis’s geographical variations.

Olędzki and Harasym (contribution 3) examined the impact of heat treatments on the bioactive properties and color–structural characteristics of bell peppers at different maturity stages (green, yellow, and red). Heat treatments such as contact grilling and roasting combined with microwaving increased the total phenolic content (TPC) of green peppers, while roasting and steaming decreased the antioxidant activity of yellow bell peppers. Moreover, certain methods significantly reduced the content of reducing sugars in red bell peppers. The study underscored the importance of selecting appropriate heat treatments to preserve the antioxidant and bioactive properties of bell peppers, thus enhancing the digestibility and bioavailability of these bioactive constituents.

Sousa et al. (contribution 4) investigated how different time/temperature combinations (65 °C/30 min; 77 °C/1 min; 88 °C/15 s; and 120 °C/20 min) affect the chemical composition and microbial load of olive pomace paste, a by-product of olive oil production. They found that while there were significant changes in components like ash, fat, vitamin E, TPC, total flavonoid content (TFC), and hydroxytyrosol and in antioxidant activity, the fatty acid profile remained constant. The 88 °C/15 s combination was the most effective in preserving the beneficial properties of the paste, making it a feasible industrial-scale solution to provide a safe and sustainable functional ingredient for novel food products.

In a different study, Ramarao et al. (contribution 5) explored the effects of convective air-drying, oven-drying, and sun-drying methods on the antioxidant properties of betel (*Piper betle* L.) leaves, with fresh leaves serving as a control. The findings revealed that sun-dried leaves exhibited superior antioxidant properties, including better total antioxidant and DPPH radical scavenging activities and higher TPC and TFC, along with a lower alkaloid concentration. Additionally, aqueous extracts from fresh and sun-dried leaves contained constituents with antioxidant and anti-inflammatory effects. These findings suggest that sun-drying maintains the antioxidant potential of betel leaves while augmenting the presence of biologically active phytoconstituents, suggesting potential applications in the food and pharmaceutical industries. Further research was recommended to assess safety aspects and individual composition variations.

Núñez-Gómez et al. (contribution 6) explored the chemical composition, functional properties, and antioxidant capacity of dietary fiber extracted from lemon peel by drying with warm air and enzymatic hydrolysis with pectinesterase. The enzymatic treatment caused a reduction in soluble fiber and an increase in insoluble fiber, along with changes in the pectin structure, resulting in diminished water and fat absorption capacities; it also decreased the TPC and antioxidant activity. Conversely, warm air drying had higher potential for producing high-quality fiber with antioxidant properties from lemon peel. These findings hold significant implications for the development of novel ingredients rich in dietary fiber and (poly)phenols with antioxidant capacity, as well as for the valorization of lemon peels and mitigation of the environmental impact associated with their disposal.

The study by dos Santos et al. (contribution 7) explored the in vitro antidiabetic, antiglycation, and antioxidant properties of an ethanolic seed extract of *Passiflora edulis* and piceatannol. Both samples significantly inhibited the α-amylase, α-glucosidase, and dipeptidyl-peptidase-4 enzymes, with IC_50_ values indicating their effectiveness compared to standard drugs. The formation of advanced glycation end-products (AGEs) and β-amyloid fibrils was also inhibited, demonstrating antiglycation properties. The samples also showed strong antioxidant activity. These findings suggested the potential of both *P. edulis* seed extract and piceatannol as antidiabetic agents, warranting further investigation.

The study conducted by Maliar et al. (contribution 8) addressed the need for a better understanding of both antioxidant and pro-oxidant effects. A methodology for the simultaneous determination of antioxidant and pro-oxidant activity on a single microplate was developed, assuming that the FRAP method could measure both effects due to the generation of pro-oxidant Fe^2+^ ions in the Fenton reaction. The study suggested that compounds with higher numbers of oxygen heteroatoms, particularly sp2-hybridized compounds like flavonoids, exhibit dominant pro-oxidant effects. Conversely, catechins, carotenoids, and certain plant extracts, such as those from birch and chestnut leaves, demonstrate dominant antioxidant activity over pro-oxidant. These initial findings prompt further systematic evaluation of a broader range of compounds and plant extracts using this method.

The potential of a standardized botanical composition from *Scutellaria baicalensis* and *Acacia catechu* in mitigating acute inflammatory lung injury and reducing mortality by reducing extracellular HMGB1 levels was investigated by Yimam et al. (contribution 9). HMGB1 is a crucial late inflammatory mediator associated with air pollution-induced oxidative stress and lung injury. The botanical composition was tested using murine models of acute lung injury and sepsis. Significant reductions in mortality, pro-inflammatory cytokines and chemokines, bacterial counts in the lungs and airways, and extracellular HMGB1 levels were observed. Moreover, cultured macrophages treated with the botanical product exhibited increased phagocytic activity and decreased extracellular HMGB1 levels. These findings suggested that the botanical could potentially protect against oxidative stress-induced lung damage by reducing extracellular HMGB1 accumulation.

Regarding in vivo studies, Truong et al. (contribution 10) investigated the protective effects of orange sweet pepper (*Capsicum annuum* L.) juices, prepared by both high-speed blender and low-speed masticating juicer, against UVB-induced skin damage in SKH-1 hairless mice. Oral administration of these juices reduced UVB-induced skin photoaging by regulating genes involved in dermal matrix production and maintenance, such as collagen type I α 1 and matrix metalloproteinases-2, 3, and 9. The juices also restored total collagen levels in UVB-exposed mice and downregulated the expression of pro-inflammatory proteins, including cyclooxygenase-2, interleukin (IL)-1β, IL-17, and IL-23, likely via inhibiting the NF-κB pathway. Additionally, the juices enhanced primary antioxidant enzymes in the skin, such as catalase, glutathione peroxidase, and superoxide dismutase-2, and reduced UVB-induced DNA damage by preventing 8-OHdG formation. These findings suggest that sweet pepper juices offer a protective effect against photoaging by inhibiting dermal matrix breakdown, inflammatory response, and DNA damage, while enhancing antioxidant defense, ultimately leading to a reduction in skin damage.

In another work, Yang et al. (contribution 11) investigated the protective effects and mechanisms of salidroside in age-related renal fibrosis using SAMP8 mice. The administration of salidroside for 12 weeks led to improvements in renal function, with reduced levels of blood urea nitrogen and serum creatinine, and increased serum albumin levels. Additionally, this phenylpropanoid glycoside reduced mesangial hyperplasia and levels of transforming growth factor-β and α-smooth muscle actin, indicating the mitigation of renal fibrosis. The treatment also decreased lipid peroxidation in the kidneys and regulated iron transport-related proteins and ferroptosis-related proteins. These findings suggested that salidroside delays renal aging and inhibits age-related glomerular fibrosis by suppressing ferroptosis in SAMP8 mice.

Lastly, the study by Mattioli et al. (contribution 12) investigated the impact of dietary *n*-3 polyunsaturated fatty acids (PUFA) on apelin and resolvin D1 (RvD1) levels in rabbit testis and sperm. Apelin is an endogenous peptide known for its involvement in both the release of inflammatory mediators and the expression of antioxidant enzymes, whereas RvD1 is a specialized pro-resolving mediator derived from *n*-3 PUFA [3]. The authors fed rabbits diets enriched with either flaxseed (rich in α-linolenic acid) or fish oil (containing eicosapentaenoic acid, docosapentaenoic acid, and docosahexaenoic acid) and observed increased apelin levels in testes of both groups, particularly in the interstitial tissue of the flaxseed-fed rabbits. The flaxseed-enriched diet also enhanced serum testosterone levels, while both diets led to higher malondialdehyde and RvD1 levels in the testis. In ejaculated sperm, apelin and RvD1 were mainly located in the tail, with positive correlations observed between apelin, sperm motility, and RvD1 levels, suggesting their potential involvement in male reproduction and inflammation resolution. These findings underscore the potential benefits of a flaxseed-enriched diet in increasing testicular apelin levels, thereby potentially ameliorating male reproductive health and inflammatory status.

## 3. Conclusions

The articles comprising this Special Issue highlight the pivotal role of antioxidants in promoting human health and well-being, alongside their versatile applications across diverse industrial sectors such as food, pharmaceuticals, and medicine. Through deeper exploration of antioxidant compounds from different foods and natural products and their underlying mechanisms of action, these studies pave the way for leveraging their therapeutic and technological potential in combating oxidative stress and its related diseases. Furthermore, this advancement will enable the development of innovative strategies for the preservation and functionalization of foods and dietary supplements, with the ultimate goal of improving the overall quality of life for individuals worldwide.
